# Trends in Use of Daily Chest Radiographs Among US Adults Receiving Mechanical Ventilation

**DOI:** 10.1001/jamanetworkopen.2018.1119

**Published:** 2018-08-10

**Authors:** Hayley B. Gershengorn, Hannah Wunsch, Damon C. Scales, Gordon D. Rubenfeld

**Affiliations:** 1 Division of Pulmonary, Allergy, Critical Care, and Sleep Medicine, Department of Medicine, Leonard M. Miller School of Medicine, University of Miami, Miami, Florida; 2 Division of Critical Care Medicine, Department of Medicine, Albert Einstein College of Medicine, Bronx, New York; 3 Department of Anesthesiology, Columbia University Medical College, New York, New York; 4 Department of Anesthesia, University of Toronto, Toronto, Ontario, Canada; 5 Interdepartmental Division of Critical Care Medicine, University of Toronto, Toronto, Ontario, Canada; 6 Department of Critical Care Medicine, Sunnybrook Health Sciences Centre, Toronto, Ontario, Canada; 7 Department of Medicine, University of Toronto, Toronto, Ontario, Canada

## Abstract

**Importance:**

Guidelines from December 2011 recommended against obtaining daily chest radiographs (CXRs) for patients requiring mechanical ventilation (MV). Daily CXR use for patients receiving MV in US hospitals is unknown and, if high, may represent an opportunity to reduce low-value care and unnecessary radiation.

**Objectives:**

To determine frequency of daily CXR use for US patients receiving MV, assess variability across hospitals, and evaluate whether use has decreased over time.

**Design, Setting, and Participants:**

Retrospective cohort study of hospitalized adults (aged ≥18 years) receiving MV for 3 days or longer. Mechanical ventilation was defined by having an *International Classification of Diseases, Ninth Revision, Clinical Modification* code of 96.7x and an MV charge on more than 1 hospital day. Hospital discharges in the Premier Perspectives database were examined from July 1, 2008, to December 31, 2014. Data analysis was conducted from July 28, 2017, to December 13, 2017.

**Exposures:**

Hospital discharge date (quarter of the year) and hospital in which patients received MV.

**Main Outcomes and Measures:**

The outcome was daily CXR use (up to 7 days) during MV. We used standard statistics to describe CXR use, multilevel multivariable regression modeling with adjusted median odds ratio (OR) to evaluate variability by hospital, and multivariable piecewise regression (breakpoint: fourth quarter of 2011) with adjusted OR to evaluate time trends and response to guideline recommendations.

**Results:**

The primary cohort included 512 518 patients receiving MV (mean [SD] age, 63.0 [16.1] years; 46% female) in 416 hospitals, of whom 321 093 (63%) received daily CXRs. Wide variability was seen across hospitals; hospitals performed daily CXRs on a median of 66% of patients (interquartile range, 50%-77%; full range, 12%-97%). The adjusted median OR was 2.43 (95% CI, 2.29-2.59), suggesting the same patient had 2.43-fold higher odds of receiving a daily CXR if admitted to a higher- vs lower-use hospital; the odds of receiving daily CXRs were unchanged through quarter 3 of 2011 (adjusted OR, 1.00; 95% CI, 0.99-1.01), after which there was a 3% relative reduction in the odds of daily CXR use per quarter (adjusted OR, 0.97; 95% CI, 0.96-0.98).

**Conclusions and Relevance:**

Three-fifths of US patients receiving MV also received daily CXRs from 2008 to 2014, although use declined slowly after new guidelines were published. The hospital at which a patient received care was associated with the odds of daily CXR receipt.

## Key Points

**Question:**

How does the prevalence of routine daily chest radiography in mechanically ventilated patients hospitalized in the United States—a practice that is no longer recommended—vary across hospitals and over time?

**Findings:**

In this cohort study of 512 518 patients receiving mechanical ventilation, 63% received a chest radiograph every day up to 7 days following mechanical ventilation initiation. The odds of receiving a daily chest radiograph were 2.43-fold higher if the same patient was discharged from a higher- vs lower-use hospital and, starting in the fourth quarter of 2011, there was a 3% relative reduction in the odds of daily use per quarter through 2014.

**Meaning:**

Mechanically ventilated patients in US hospitals continue to receive chest radiographs daily at high rates even though guidelines recommend against this practice; use depends largely on the hospital at which the patient receives care rather than individual patient characteristics.

## Teaser

This cohort study determines the frequency of daily chest radiograph use for adult patients receiving mechanical ventilation in the United States, assesses variability across hospitals, and evaluates whether use has decreased over time.

## Introduction

Routine daily chest radiographs (CXRs) were once a common practice in US intensive care units (ICUs) bolstered by early studies associating CXR findings with clinical management decisions.^1,2,3,4,5,6,7,8^ The American College of Radiology (ACR) assigned daily portable CXRs its “most appropriate” rating for patients receiving mechanical ventilation (MV) up until 2008.^9,10^ However, later work revealed that while therapeutic decisions may be affected by CXR results, patient-centered outcomes (eg, mortality, length of stay, and duration of MV) are not associated with routine daily CXRs.^11,12,13,14,15,16,17,18,19^ Citing this evidence base, the ACR amended its recommendations in December 2011 by assigning a “usually not appropriate” rating with some exceptions to routine daily CXRs.^20^ In 2014, the entire category of patients receiving MV were removed and routine CXRs in all stable patients in the ICU were categorized as “usually not appropriate.”^21,22^ Also in that year, the 4 major US critical care professional societies endorsed “Do not order diagnostic tests at regular intervals (such as every day)”, specifically including daily CXRs as the first recommendation of their Choosing Wisely top 5 list.^23^

Little is known about real-world use of routine CXRs. A single-day snapshot of 854 CXRs obtained from 804 patients in 104 French ICUs in 2012 revealed that only 37% were ordered as part of routine care,^24^ which is consistent with a 2008 survey of French intensivists that found only 25% supported routine CXRs for patients receiving MV.^25^ There are limited data on the evolution of practice over time. Two surveys from Dutch ICUs in 2005 and 2013 with different response rates found a decrease from 63% to 7% of ICUs reporting the use of routine daily CXRs.^26,27^ To our knowledge, there are no studies of practice in the United States to evaluate the use of routine daily CXRs.

We conducted a retrospective cohort analysis of adults receiving MV in US hospitals from 2008 to 2014. We hypothesized that, after adjustment for patient case mix and hospital factors, the use of daily CXRs remains high, varies widely from hospital to hospital, and has been decreasing over time in response to a growing evidence base and ACR guideline recommendations. Understanding trends in daily CXR use can elucidate whether interventions aimed at limiting this low-value practice are needed. Moreover, finding significant interhospital variability unexplained by case mix suggests that institutional factors such as culture and teamwork should be addressed to facilitate widespread de-adoption.

## Methods

We conducted a retrospective cohort study of patients discharged from hospitals in the Premier Perspectives database from July 1, 2008, through December 31, 2014. Premier Perspectives is a large US database including approximately 20% of all US hospital discharges from more than 700 hospitals.^28^ Premier, Inc provides audit feedback to participating hospitals. Participation is voluntary and hospitals pay for the service. This study follows the Strengthening the Reporting of Observational Studies in Epidemiology (STROBE) reporting guideline. This study was exempted from review by the institutional review board at the University of Miami Miller School of Medicine.

Available data on hospital characteristics include number of beds, region (Midwest, Northeast, South, and West of the United States), community (rural vs urban), and teaching status. Demographic data include age, race (white, black, or other), sex, marital status, and primary source of payment for medical services. Chronic medical conditions were identified using *International Classification of Diseases, Ninth Revision, Clinical Modification* (*ICD-9-CM*) codes and diagnostic-related groups as described by Elixhauser et al.^29^ Major surgery was defined using *ICD-9-CM* procedure codes as classified by the Healthcare Cost and Utilization Project.^30^ Major diagnostic categories^31^ were used to classify admission diagnoses. Daily charges data were used to identify timing of invasive MV^32^ and timing of CXRs (eTable 1 in the Supplement).

**Figure 1.**
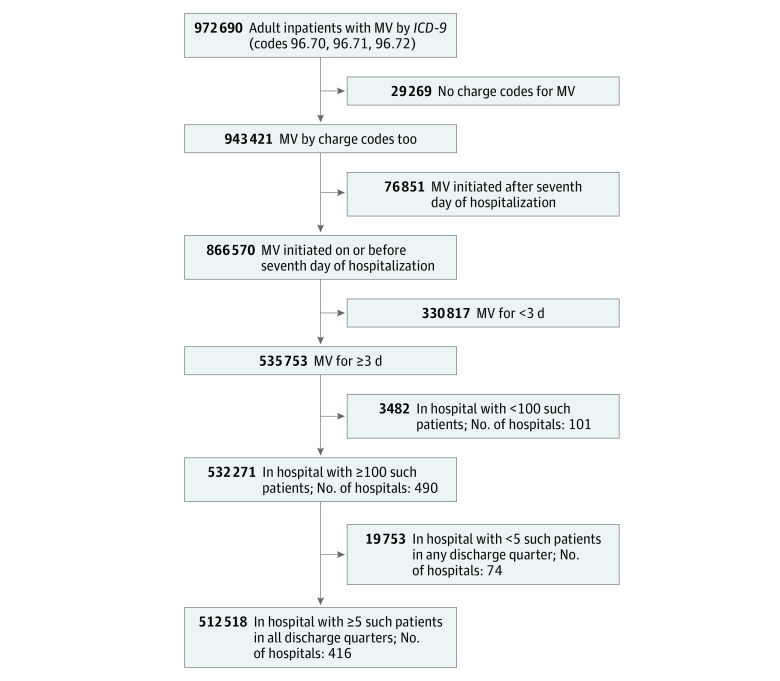
Flow Diagram *ICD-9* indicates *International Classification of Diseases, Ninth Revision*; MV, mechanical ventilation.

Our primary cohort consisted of hospitalized adults (aged ≥18 years) receiving invasive MV (*ICD-9-CM* codes 96.7x + charges indicating MV^32^ on ≥1 hospital day) on at least 3 consecutive days during their first episode of MV starting on or before their seventh day of hospitalization (Figure 1). The requirement of at least 3 calendar days of MV ensured we did not include patients in whom only the initial day of MV would be considered as eligible for CXR receipt as a CXR to confirm endotracheal tube placement is recommended.^9,10,20,21,22^ To avoid including hospitals where MV use was infrequent, patients were excluded if they were admitted to a hospital that contributed fewer than 100 such patients to the data set over the entire period and/or fewer than 5 such patients during any discharge quarter (3-month period).

The primary outcome, daily CXR use, was defined as the receipt of a CXR on every day that a patient received MV for up to 7 days following MV initiation (day 1 = day of MV initiation); the final day of MV was not considered (eFigure 1 in the Supplement). All patients with MV durations of 8 days or more were evaluated for CXR receipt on only their first 7 days following MV initiation.

Sensitivity analyses using 5 alternative cohort definitions were performed to test the robustness of our findings, which included: (1) only patients with MV durations of 8 days or more (requiring exactly 7 CXRs to meet definition of receiving daily CXRs); (2) excluding patients who received MV but did not have charges for care in an ICU; (3) excluding patients with chest tubes (as determined by charge codes; eTable 1 in the Supplement); (4) excluding patients admitted for cardiac surgery (as determined by Medicare Severity–Diagnosis Related Groups^33,34^); and (5) excluding hospitals where more than 10% of patients receiving MV were cardiac surgery patients. Cohort 1 was used to evaluate whether de-adoption was more rapid in patients requiring prolonged MV. Cohort 2 was used to account for the unusual scenario observed in some hospitals where patients were mechanically ventilated without codes suggesting care in an ICU. Cohorts 3 through 5 were used to exclude patients where daily CXRs might have been used to assess other intrathoracic devices.

### Statistical Analysis

Standard summary statistics were used to report baseline characteristics and unadjusted outcomes for the entire cohort as well as for patients stratified by receipt of a daily CXR. Tests used were χ^2^, *t*, and Kruskal-Wallis to compare characteristics and outcomes between groups as appropriate.

Unadjusted variability in daily CXR use was assessed across individual hospitals by comparison of the proportion of patients receiving a daily CXR. A multilevel multivariable logistic regression model (with clustering by hospital to allow for random intercepts) was constructed to adjust for patient- and hospital-level factors potentially associated with daily CXR use (modeled as fixed effects); all available patient- and hospital-level variables as well as quarter of hospital discharge were included in the model as covariates. Adjusted median odds ratios (ORs) were used to quantify the effects of hospital practice patterns by estimating the odds of receiving a CXR if the same patient were admitted to a hospital with higher vs lower rates of daily CXR use; specifically, the median OR is constructed by examining all possible pairs of hospitals in the full cohort and taking the median of the increased odds of daily CXR use at the higher- vs the lower-use hospital within each pair after adjustment for covariates.^35,36,37^ To further estimate the hospital effect, we compared patients admitted with hospitals in the highest vs lowest quartiles of daily CXR use with similar univariable and multivariable techniques. Discharge quarter was included in these models as a linear covariate.

Unadjusted trends in practice were explored using proportions of patients receiving a daily CXR across hospital discharge quarters. The adjusted association of discharge quarter and daily CXR use was first assessed using the multilevel multivariable logistic regression model. To assess the possible association of hospital-level factors and trends in daily CXR use over time, we also sequentially constructed 4 models, each with an interaction term of discharge quarter and 1 of the 4 available hospital-level variables (number of beds, region, community, and teaching status); statistically significant interactions (*P* < .05) were considered associated with trends in practice.

We constructed a piecewise regression model to evaluate the effect of the ACR guidelines change that was made public in December 2011 (fourth quarter [Q4] of 2011). First, we confirmed the use of Q4 of 2011 as an appropriate breakpoint using least-squares univariable regression (allowing all values for discharge quarter to be considered as the breakpoint, as well as different regression coefficients for the association of discharge quarter with daily CXR use before and after the best-fit breakpoint to be considered). Second, we constructed a piecewise multivariable regression model using a breakpoint of Q4 of 2011, the possibility of 2 unique trends with time before and after Q4 of 2011, and the possibility for a step change at Q4 of 2011. This model was not chosen as our primary model as it did not converge in a multilevel format; as such, it is less useful to assess variability at the individual hospital level.

Multivariable multilevel models including discharge quarter as a linear covariate (similar to our primary model) were then repeated for each sensitivity cohort. When multilevel models did not converge, single-level multivariable regression models with clustering of standard errors by individual hospital were used.

All statistical analyses were performed using Stata, version 15 (StataCorp LLC); SAS, version 9.4 (SAS Institute Inc); and Microsoft Excel (Microsoft Corp). Comparisons and associations were considered statistically significant if 2-sided *P* < .05.

## Results

The primary cohort consisted of 512 518 patients receiving MV (mean [SD] age, 63.0 [16.1] years; 46% female) in 416 hospitals, of whom 321 093 (63%) received a CXR every day up to 7 days following MV initiation (Figure 1 and Table). Characteristics of patients who received a daily CXR were statistically significantly different from patients who did not receive daily CXRs (*P* < .001 for all comparisons). Patients who received daily CXRs vs no daily CXRs more commonly were white (214 208 [66.7%] vs 118 905 [62.1%]), were privately insured (59 977 [18.7%] vs 31 726 [16.6%]), were admitted with major surgery (99 487 [31.0%] vs 50 794 [26.5%]), had a cardiovascular admitting diagnosis (44 473 [13.9%] vs 17 572 [9.2%]), and had shorter duration of MV (median [interquartile range], 4 [2-8] vs 6 [4-11] days); similar associations with patient-level factors were seen after multivariable adjustment (eTable 2 in the Supplement). Patients with daily CXRs vs no daily CXRs were less frequently admitted to teaching hospitals (147 431 [45.9%] vs 95 158 [49.7%]); this association was not maintained after multivariable adjustment.

**Table.** Baseline Characteristics and Unadjusted Outcomes Stratified by Daily CXR Use^a^

Characteristics	No. (%)		
	Full Cohort	No Daily CXR	Daily CXR
Patient characteristics			
No. of patients	512 518	191 425 (37.3)	321 093 (62.7)
Age, y			
<50	96 904 (18.9)	35 834 (18.7)	61 070 (19.0)
50-64	160 773 (31.4)	60 644 (31.7)	100 129 (31.2)
65-84	214 179 (41.8)	79 519 (41.5)	134 660 (41.9)
≥85	40 662 (7.9)	15 428 (8.1)	25 234 (7.9)
Female^b^	235 944 (46.0)	90 801 (47.4)	145 143 (45.2)
Race			
White	333 113 (65.0)	118 905 (62.1)	214 208 (66.7)
Black	82 441 (16.1)	35 227 (18.4)	47 214 (14.7)
Other	96 964 (18.9)	37 293 (19.5)	59 671 (18.6)
Insurance			
Private	91 703 (17.9)	31 726 (16.6)	59 977 (18.7)
Medicare	300 818 (58.7)	113 929 (59.5)	186 889 (58.2)
Medicaid	71 309 (13.9)	28 701 (15.0)	42 608 (13.3)
Other or unknown	48 688 (9.5)	17 069 (8.9)	31 619 (9.8)
Elixhauser comorbidities, median (IQR), No.	5 (3-6)	5 (3-6)	5 (3-6)
Major surgery	150 281 (29.3)	50 794 (26.5)	99 487 (31.0)
Major diagnostic category^c^			
Could not be assigned	75 902 (14.8)	31 261 (16.3)	44 641 (13.9)
Nervous system	44 217 (8.6)	19 253 (10.1)	24 964 (7.8)
Respiratory	125 876 (24.6)	45 459 (23.7)	80 417 (25.0)
Cardiovascular	62 045 (12.1)	17 572 (9.2)	44 473 (13.9)
Gastrointestinal (nonliver)	24 331 (4.8)	9368 (4.9)	14 963 (4.7)
Liver	9425 (1.8)	3542 (1.9)	5883 (1.8)
Musculoskeletal	8635 (1.7)	2935 (1.5)	5700 (1.8)
Endocrine	4499 (0.9)	1741 (0.9)	2758 (0.9)
Renal	7556 (1.5)	3436 (1.8)	4120 (1.3)
Infectious	110 677 (21.6)	43 615 (22.8)	67 062 (20.9)
Alcohol and/or drug use	2513 (0.5)	0948 (0.5)	1565 (0.5)
Injuries and/or poisonings	17 813 (3.5)	5674 (3.0)	12 139 (3.8)
Multiple trauma	7145 (1.4)	1814 (0.9)	5331 (1.7)
HIV	2530 (0.5)	946 (0.5)	1584 (0.5)
Discharge, y			
2008	28 447 (5.6)	9672 (5.1)	18 775 (5.8)
2009	67 878 (13.2)	23 142 (12.1)	44 736 (13.9)
2010	74 112 (14.5)	25 634 (13.4)	48 478 (15.1)
2011	82 936 (16.2)	28 917 (15.1)	54 019 (16.8)
2012	86 254 (16.8)	31 790 (16.6)	54 464 (17.0)
2013	92 618 (18.1)	37 481 (19.6)	55 137 (17.2)
2014	80 273 (15.7)	34 789 (18.2)	45 484 (14.2)
Hospital characteristics			
No. of hospital beds			
≥500	178 244 (34.8)	67 998 (35.5)	110 246 (34.3)
400-499	92 560 (18.1)	35 715 (18.7)	56 845 (17.7)
300-399	101 251 (19.8)	35 638 (18.6)	65 613 (20.4)
200-299	85 847 (16.8)	30 769 (16.1)	55 078 (17.2)
100-199	50 382 (9.8)	20 241 (10.6)	30 141 (9.4)
<100	4234 (0.8)	1064 (0.6)	3170 (1.0)
Teaching hospital	24 589 (47.3)	95 158 (49.7)	147 431 (45.9)
Urban hospital	463 430 (90.4)	175 222 (91.5)	288 208 (89.8)
Geographic region			
Midwest	97 108 (19.0)	34 738 (18.1)	62 370 (19.4)
Northeast	91 558 (17.9)	38 836 (20.3)	52 722 (16.4)
South	243 126 (47.4)	88 968 (46.5)	154 158 (48.0)
West	80 726 (15.8)	28 883 (15.1)	51 843 (16.1)
Outcomes			
MV duration, median (IQR), d	5 (3-9)	6 (4-11)	4 (2-8)
Hospital length of stay, median (IQR), d	12 (7-19)	12 (8-20)	12 (7-19)
Hospital mortality	137 265 (26.8)	52 356 (27.4)	84 909 (26.5)

Abbreviations: CXR, chest radiograph; IQR, interquartile range; MV, mechanical ventilation.

^a^ All comparisons by daily CXR status (*P* < .001 [χ^2^ testing for categorical and Kruskal-Wallis testing for continuous variables]).

^b^ Missing from 47 patients (0.009%) in the full cohort.

^c^ None from neonatal; less than 0.5% from eye, ear, nose, and throat, dermatologic, genitourinary (male), genitourinary (female), obstetric, hematologic, myeloproliferative, psychiatric, burns, and health status influences.

**Figure 2.**
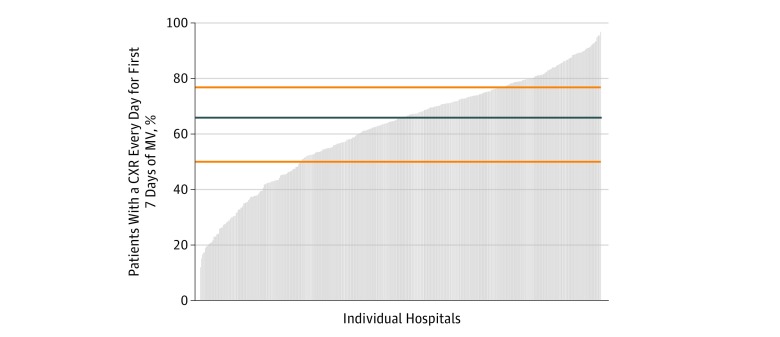
Percentage of Patients Receiving a Chest Radiograph (CXR) Every Day (Up to 7 days) Following Mechanical Ventilation (MV) Initiation Stratified by Individual Hospital Each gray bar indicates an individual hospital; blue line, median value across all hospitals; and orange lines, 25th and 75th percentile values across all hospitals.

**Figure 3.**
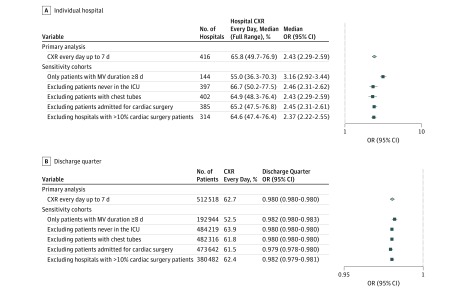
Adjusted Association of Individual Hospital and Discharge Quarter With the Receipt of a Chest Radiograph (CXR) Every Day Following Mechanical Ventilation (MV) Initiation All models are multilevel multivariable logistic regression except for the primary cohort, CXR every day up to 14 days, for which the model did not converge and a single-level multivariable logistic regression model with clustering of standard errors by hospital was used. Covariates adjusted for in addition to discharge quarter (modeled as a linear predictor) include age, sex, race, insurance provider, number of Elixhauser comorbidities, major surgery, major diagnostic category, hospital bed number, hospital teaching status, urban hospital, and geographic region. Cardiac surgery was defined using Medicare Severity–Diagnosis Related Group (those suggested by Center for Medicare and Medicaid Services; 216-244, 246-265,^33^ 215, and 245). On forest plots, markers indicate point estimates; error bars, 95% confidence intervals. The x-axis is on a logarithmic scale. ICU indicates intensive care unit; OR, odds ratio.

Wide variability in daily CXR use was seen across individual hospitals (Figure 2); hospitals performed daily CXRs on a median of 66% of patients (interquartile range, 50%-77%; full range, 12%-97%). After multivariable adjustment, the median OR associated with individual hospital of admission was 2.43 (95% CI, 2.29-2.59; Figure 3; eTable 2 in the Supplement) suggesting that the same patient had 2.43-fold higher odds of receiving a daily CXR when admitted to a higher- vs lower-use hospital. The only hospital-level factor found to be independently associated in the multivariable model with higher hospital-level daily CXR use was being located in the Northeastern United States (OR, 0.27; 95% CI, 0.07-0.96; *P* = .04) compared with the Midwest region (eTable 3 in the Supplement).

**Figure 4.**
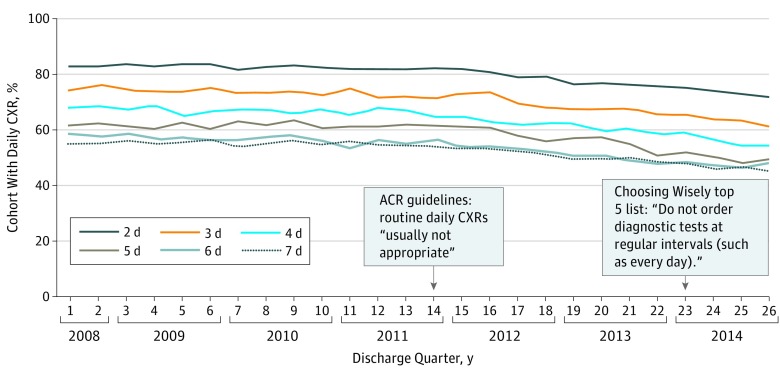
Percentage (Unadjusted) of Patients Receiving a Chest Radiograph (CXR) Every Day (Up to 7 days) Following Mechanical Ventilation (MV) Initiation by Discharge Quarter Trends in daily CXR use over time were not statistically significantly different across MV duration groups, with *P* = .22 to .62 for the interaction of MV duration and discharge quarter in a model with percentage of daily CXR use as the independent variable and MV duration, discharge quarter, and their interaction as the dependent variables. ACR indicates American College of Radiology.

Daily CXR use declined over time from 66% of patients in the third quarter of 2008 to 56% in Q4 of 2014 (Figure 4). This decline in frequency of daily CXRs was not limited to patients with longer durations of MV; it was experienced by all patients (*P* = .22 to .62 for interactions of each MV duration and discharge quarter). Modeling a stable trend from 2008 to 2014, after multivariable adjustment, there was a 2% relative reduction in the odds of receiving a daily CXR per discharge quarter (OR, 0.98; 95% CI, 0.98-0.98) (Figure 3; eTable 2 in the Supplement). A significant interaction was found between 2 hospital-level factors (hospital size and geographical location) and the odds of receiving a daily CXR (eFigure 2 in the Supplement). Very small hospitals (<100 beds) had the highest rates of daily CXR use at the start of the study period, but experienced the largest decline in use over time. Hospitals in the West only began de-adopting daily CXR use in the last year of the study period, whereas those located elsewhere experienced a steadier decline over time; hospitals in the Northeast used daily CXRs less frequently throughout the study period. No interaction was found between teaching status or urban location and the odds of receiving a daily CXR.

Univariate regression confirmed Q4 of 2011—the time of change in ACR recommendations—as the best-fit breakpoint. There was no change in the odds of daily CXR use prior to Q4 of 2011 (annual OR, 1.00; 95% CI, 0.99-1.01), but there was a 3% relative reduction in the odds of daily CXR use per quarter starting in Q4 of 2011 (OR, 0.97; 95% CI, 0.96-0.98); there was no step change in use at Q4 of 2011 (eFigure 3 in the Supplement).

Our results were robust across our 5 sensitivity cohorts. Adjusted median ORs (indicating hospital-level variability in daily CXR use) were similar to the results obtained in the primary analysis (Figure 3). Moreover, in each of the 5 additional analyses, we found the same 2% relative reduction in the odds of daily CXR use per discharge quarter (modeled as a stable trend from 2008-2014).

## Discussion

Despite recommendations against performing CXRs on a daily basis, we found that three-fifths of patients received a CXR every day up to 7 days following MV initiation in the United States. Practice varied substantially across hospitals, but three-quarters of hospitals performed daily CXRs on at least half of their patients receiving MV. The practice of obtaining daily CXRs, after adjustment for patient case mix and hospital factors, declined from 2008 to 2014 with a notable, although small, change from a 0% to a 3% relative reduction in the odds of use per quarter after the ACR updated their recommendations in 2011. Despite this change in practice, more than half of our cohort of patients receiving MV continued to receive daily CXRs in 2014. Even after adjusting for patient variables, the hospital in which the patient received MV was the strongest risk factor for whether daily CXRs were obtained—higher-use hospitals were more than twice as likely to order daily CXRs.

Our study cannot determine why rates of daily CXR use remain high despite recommendations to the contrary from the ACR and the Choosing Wisely campaign. Possible explanations are that clinicians agree with the recommendations against daily CXR use but are incentivized not to follow them; clinicians agree with the recommendations and would like to comply with them but have a difficult time doing so; clinicians do not agree with the recommendations; or clinicians are unaware of them. Often we must be concerned that clinicians are financially incentivized to provide low-value care with fee-for-service models that reward quantity rather than quality.^38^ However, this issue is unlikely at play here as the clinical physicians ordering CXRs do not receive financial compensation from this practice (and radiologists who are compensated are not in a position to order the studies). Instead, it is more likely that de-adoption of standard-of-care practices is challenging. Even with clear and convincing evidence to support de-adoption, frequently the critical care community has done so slowly and incompletely.^39,40,41,42^ Altering care from an automated system where CXRs can be ordered as the default to a system where every CXR requires a clinical decision both is time-consuming and can add to decision fatigue. Another explanation may be found in a key premise of implementation science—changes in practice are less likely to be implemented if they contradict the clinician’s experience.^43^ By their very nature, all attempts at de-adoption do exactly this. It is possible that clinicians simply do not agree with the recommendations. A meta-analysis of 9 randomized and quasi-randomized clinical trials including 9611 patients with 39 358 CXRs found no association between a restrictive strategy of not obtaining daily CXRs with mortality (ICU or hospital), length of stay (ICU or hospital), or duration of MV; however, as the authors note, confidence intervals were wide, leaving the “safety of abandoning routine CXRs… uncertain.”^19^ However, it is difficult to prove a negative, and some clinicians may simply be unconvinced that routine CXRs provide no benefit.

In the absence of compelling data for or against the practice, it is not surprising that daily CXR use and trends in use vary widely across individual hospitals. The risk to critically ill patients of CXRs is likely perceived to be low with little radiation exposure^44^ and no potential harm associated with transport out of the ICU. With low risk and low but potential benefit, the risk-benefit analysis may leave clinicians willing to yield to institutional practice and standardized orders regarding daily CXRs.

Widespread de-adoption of daily CXRs for patients receiving MV would provide cost savings. As an estimate, if the rates of daily CXR use in all hospitals in the highest 3 quartiles of use were decreased to the rate of the hospital with the highest rate of use in the lowest quartile (ie, only 50% of patients receiving MV in these higher-use hospitals received a daily CXR instead of 71%), more than 2 300 000 fewer CXRs would be performed in the United States annually at an estimated cost savings of more than $144 million (eMethods in the Supplement). Although the radiation dose from an individual CXR is generally considered negligible,^45^ the cumulative exposure from unnecessary diagnostic procedures to patients and staff is unjustified.

### Strengths

Strengths of this study include the large representative sample, the availability of detailed daily charges data, and the robustness of our results across several sensitivity analyses. The multilevel multivariable modeling approach, in which we adjusted for available patient and hospital factors and clustered patients within their hospitals of care, allowed us to identify time trends and hospital variability in daily CXR use, which is independent of changes in case mix or identifiable hospital characteristics.

### Limitations

As a retrospective cohort study of administrative data, despite attempts to adjust for confounding by indication and to exclude patient groups likely to have an accepted reason for obtaining a daily CXR, some patients who received daily CXRs in this cohort may have had other clinical indications. However, such residual confounding should not differ systematically across hospitals. As our cohort was derived from administrative data records and defined, in part, by *ICD-9-CM* codes, we likely underestimated the true number of patients receiving MV at each hospital.^46^ Our inclusion criteria were only moderately sensitive, but the high specificity of *ICD-9-CM* codes for MV identification and our coupling of them with billing information means that we are confident that patients categorized as receiving MV did so with a very high likelihood. Our definition of daily use (ie, including CXRs done on every day up to day 7 of MV excluding the final day of MV) is novel; it is possible that use of a differently characterized outcome variable could affect our results. While differences are noted in the comparisons of mortality and length of stay, the study was not designed to evaluate whether daily CXRs affect outcome; these comparisons are unadjusted and may reflect differences in patients or in hospitals that perform daily CXRs.

## Conclusions

Daily CXR use in patients receiving MV in the United States, while declining, remains a prevalent practice. The hospital where a patient receives MV appears to be the largest driver of use of daily CXRs. These findings are both concerning and encouraging. Some hospitals may have created protocols, incentives, or cultures that allowed or even drove decreases in daily CXR use. This provides a key insight for targeting de-adoption of this practice, suggesting that efforts should be made at understanding and intervening at the institutional level. Moreover, the remaining widespread use means there are likely some high-use hospitals in which simple interventions could dramatically affect practice—institutions in which attempts at change might represent low-hanging fruit. We must leverage these 2 hospital groups to teach us about how to reduce daily CXR use for patients receiving MV; strategies used by the critical care community to drive de-adoption in this space may be transferrable to other aspects of low-value care.

## Notes

Supplement. **eTable 1.** List of Charge Codes Used to Identify Chest Radiographs and Chest Tubes **eTable 2.** Factors Independently Associated With Receiving a Chest Radiograph Every Day (up to 7 days) Following Mechanical Ventilation Initiation **eTable 3.** Factors Associated With Being Admitted to Hospitals with the Highest vs Lowest Quartile of Use of Chest Radiographs Every Day (up to 7 days) Following Mechanical Ventilation Initiation **eFigure 1.** Pictogram of Cohort Creation and Outcome Assessment **eFigure 2.** Adjusted Percentage of Patients Receiving a Chest Radiograph Every Day (up to 7 days) Following Mechanical Ventilation Initiation by Discharge Quarter Stratified by Hospital Size and Geographic Region **eFigure 3.** Crude and Piecewise Regression-based Predicted Percentage of Patients Receiving a Chest Radiograph Every Day (up to 7 days) Following Mechanical Ventilation Initiation by Discharge Quarter **eMethods.** Methods for Estimating Hospital Cost Savings Associated with Reduced Daily CXR Use Across the US **eReference**

## References

[1] Hall JB, White SR, Karrison T Efficacy of daily routine chest radiographs in intubated, mechanically ventilated patients. Crit Care Med. 1991;19(5):-. doi:10.1097/00003246-199105000-00015 2026031

[2] Silverstein DS, Livingston DH, Elcavage J, Kovar L, Kelly KM The utility of routine daily chest radiography in the surgical intensive care unit. J Trauma. 1993;35(4):643-646. doi:10.1097/00005373-199310000-00022 8411291

[3] Fong Y, Whalen GF, Hariri RJ, Barie PS Utility of routine chest radiographs in the surgical intensive care unit: a prospective study. Arch Surg. 1995;130(7):764-768. doi:10.1001/archsurg.1995.01430070086017 7611867

[4] Brainsky A, Fletcher RH, Glick HA, Lanken PN, Williams SV, Kundel HL Routine portable chest radiographs in the medical intensive care unit: effects and costs. Crit Care Med. 1997;25(5):801-805. doi:10.1097/00003246-199705000-00015 9187599

[5] Marik PE, Janower ML The impact of routine chest radiography on ICU management decisions: an observational study. Am J Crit Care. 1997;6(2):95-98. 9172857

[6] Chahine-Malus N, Stewart T, Lapinsky SE, Utility of routine chest radiographs in a medical-surgical intensive care unit: a quality assurance survey. Crit Care. 2001;5(5):271-275. doi:10.1186/cc1045 11737902 PMC83854

[7] Quasney MW, Goodman DM, Billow M, Routine chest radiographs in pediatric intensive care units. Pediatrics. 2001;107(2):241-248. doi:10.1542/peds.107.2.241 11158453

[8] Graat ME, Choi G, Wolthuis EK, The clinical value of daily routine chest radiographs in a mixed medical-surgical intensive care unit is low. Crit Care. 2006;10(1):R11. doi:10.1186/cc3955 16420655 PMC1550788

[9] Aquino SL, Khan A, Batra PV, ; American College of Radiology ACR Appropriateness Criteria: Routine Chest Radiograph. http://www.dcamedical.com/pdf/appropriateness-criteria-routine-chest-xray.pdf. Accessed June 18, 2017.

[10] Amorosa JK, Bramwit MP, Khan AR, ; American College of Radiology ACR Appropriateness Criteria: Intensive Care Unit Patients. Reston, VA: American College of Radiology; 2008. 10.1016/j.jacr.2012.11.013 23571057

[11] Krivopal M, Shlobin OA, Schwartzstein RM Utility of daily routine portable chest radiographs in mechanically ventilated patients in the medical ICU. Chest. 2003;123(5):1607-1614. doi:10.1378/chest.123.5.1607 12740281

[12] Hendrikse KA, Gratama JW, Hove Wt, Rommes JH, Schultz MJ, Spronk PE Low value of routine chest radiographs in a mixed medical-surgical ICU. Chest. 2007;132(3):823-828. doi:10.1378/chest.07-1162 17873192

[13] Graat ME, Kröner A, Spronk PE, Elimination of daily routine chest radiographs in a mixed medical-surgical intensive care unit. Intensive Care Med. 2007;33(4):639-644. doi:10.1007/s00134-007-0542-1 17333118 PMC1915596

[14] Mets O, Spronk PE, Binnekade J, Stoker J, de Mol BA, Schultz MJ Elimination of daily routine chest radiographs does not change on-demand radiography practice in post-cardiothoracic surgery patients. J Thorac Cardiovasc Surg. 2007;134(1):139-144. doi:10.1016/j.jtcvs.2007.02.029 17599499

[15] Clec’h C, Simon P, Hamdi A, Are daily routine chest radiographs useful in critically ill, mechanically ventilated patients? a randomized study. Intensive Care Med. 2008;34(2):264-270. doi:10.1007/s00134-007-0919-1 17994222

[16] Sy E, Luong M, Quon M, Implementation of a quality improvement initiative to reduce daily chest radiographs in the intensive care unit. BMJ Qual Saf. 2016;25(5):379-385. doi:10.1136/bmjqs-2015-004151 26350068

[17] Resnick S, Inaba K, Karamanos E, Clinical relevance of the routine daily chest x-ray in the surgical intensive care unit. Am J Surg. 2017;214(1):19-23. doi:10.1016/j.amjsurg.2016.09.059 27769542

[18] Oba Y, Zaza T Abandoning daily routine chest radiography in the intensive care unit: meta-analysis. Radiology. 2010;255(2):386-395. doi:10.1148/radiol.10090946 20413752

[19] Ganapathy A, Adhikari NK, Spiegelman J, Scales DC Routine chest x-rays in intensive care units: a systematic review and meta-analysis. Crit Care. 2012;16(2):R68. doi:10.1186/cc11321 22541022 PMC3681397

[20] Amorosa JK, Bramwit MP, Mohammed TL, ACR appropriateness criteria routine chest radiographs in intensive care unit patients. J Am Coll Radiol. 2013;10(3):170-174. doi:10.1016/j.jacr.2012.11.013 23571057

[21] Suh RD, Genshaft SJ, Kirsch J, ; American College of Radiology ACR Appropriateness Criteria: Intensive Care Unit Patients. https://acsearch.acr.org/docs/69452/Narrative/. Accessed June 17, 2018

[22] Suh RD, Genshaft SJ, Kirsch J, ACR Appropriateness Criteria® Intensive Care Unit Patients. J Thorac Imaging. 2015;30(6):W63-W65. doi:10.1097/RTI.0000000000000174 26439890

[23] Halpern SD, Becker D, Curtis JR, ; Critical Care Societies Collaborative Five things physicians and patients should question. http://www.choosingwisely.org/doctor-patient-lists/critical-care-societies-collaborative-critical-care/. Accessed June 17, 2018.

[24] Lakhal K, Serveaux-Delous M, Lefrant JY, Capdevila X, Jaber S; AzuRéa Network for the RadioDay Study Group Chest radiographs in 104 French ICUs: current prescription strategies and clinical value (the RadioDay study). Intensive Care Med. 2012;38(11):1787-1799. doi:10.1007/s00134-012-2650-9 23011527

[25] Hejblum G, Ioos V, Vibert JF, A web-based Delphi study on the indications of chest radiographs for patients in ICUs. Chest. 2008;133(5):1107-1112. doi:10.1378/chest.06-3014 17989166

[26] Graat ME, Hendrikse KA, Spronk PE, Korevaar JC, Stoker J, Schultz MJ Chest radiography practice in critically ill patients: a postal survey in the Netherlands. BMC Med Imaging. 2006;6:8. doi:10.1186/1471-2342-6-8 16848892 PMC1557847

[27] Tolsma M, Rijpstra TA, Schultz MJ, Mulder PG, van der Meer NJ Significant changes in the practice of chest radiography in Dutch intensive care units: a web-based survey. Ann Intensive Care. 2014;4(1):10. doi:10.1186/2110-5820-4-10 24708581 PMC4113284

[28] Premier, Inc Premier research services. https://www.premierinc.com/wpdm-package/research/. Accessed June 17, 2018.

[29] Elixhauser A, Steiner C, Harris DR, Coffey RM Comorbidity measures for use with administrative data. Med Care. 1998;36(1):8-27. doi:10.1097/00005650-199801000-00004 9431328

[30] Healthcare Cost and Utilization Project Procedure classes. https://www.hcup-us.ahrq.gov/toolssoftware/procedure/procedure.jsp. Accessed October 27, 2017.

[31] Centers for Medicare & Medicaid Services Draft ICD-10-CM/PCS MS-DRGv31.0 definitions manual. https://www.cms.gov/icd10manual/version31-fullcode-cms/P0001.html. Accessed October 27, 2017.

[32] Lindenauer PK, Stefan MS, Johnson KG, Priya A, Pekow PS, Rothberg MB Prevalence, treatment, and outcomes associated with OSA among patients hospitalized with pneumonia. Chest. 2014;145(5):1032-1038. doi:10.1378/chest.13-1544 24371839 PMC4011652

[33] Centers for Medicare & Medicaid Services MS-DRG surgical hierarchy by MDC and MS-DRG. https://www.cms.gov/icd10manual/fullcode_cms/P0372.html. Accessed October 27, 2017.

[34] Centers for Medicare & Medicaid Services List of MS-DRGs, version 28.0. https://www.cms.gov/icd10manual/fullcode_cms/P0368.html. Accessed October 27, 2017.

[35] Larsen K, Merlo J Appropriate assessment of neighborhood effects on individual health: integrating random and fixed effects in multilevel logistic regression. Am J Epidemiol. 2005;161(1):81-88. doi:10.1093/aje/kwi017 15615918

[36] Merlo J, Chaix B, Ohlsson H, A brief conceptual tutorial of multilevel analysis in social epidemiology: using measures of clustering in multilevel logistic regression to investigate contextual phenomena. J Epidemiol Community Health. 2006;60(4):290-297. doi:10.1136/jech.2004.029454 16537344 PMC2566165

[37] Wijeysundera DN, Austin PC, Beattie WS, Hux JE, Laupacis A Variation in the practice of preoperative medical consultation for major elective noncardiac surgery: a population-based study. Anesthesiology. 2012;116(1):25-34. doi:10.1097/ALN.0b013e31823cfc03 22185874

[38] Wang C, Kane R, Levenson M, Association between changes in CMS reimbursement policy and drug labels for erythrocyte-stimulating agents with outcomes for older patients undergoing hemodialysis covered by fee-for-service Medicare. JAMA Intern Med. 2016;176(12):1818-1825. doi:10.1001/jamainternmed.2016.6520 27775769

[39] Niven DJ, Rubenfeld GD, Kramer AA, Stelfox HT Effect of published scientific evidence on glycemic control in adult intensive care units. JAMA Intern Med. 2015;175(5):801-809. doi:10.1001/jamainternmed.2015.0157 25775163

[40] Wiener RS, Welch HG Trends in the use of the pulmonary artery catheter in the United States, 1993-2004. JAMA. 2007;298(4):423-429. doi:10.1001/jama.298.4.423 17652296

[41] Koo KK, Sun JC, Zhou Q, Pulmonary artery catheters: evolving rates and reasons for use. Crit Care Med. 2011;39(7):1613-1618. doi:10.1097/CCM.0b013e318218a045 21494107

[42] Gershengorn HB, Wunsch H Understanding changes in established practice: pulmonary artery catheter use in critically ill patients. Crit Care Med. 2013;41(12):2667-2676. doi:10.1097/CCM.0b013e318298a41e 23978814 PMC4047564

[43] Weinert CR, Mann HJ The science of implementation: changing the practice of critical care. Curr Opin Crit Care. 2008;14(4):460-465. doi:10.1097/MCC.0b013e3283079eb5 18614913

[44] Moloney F, Fama D, Twomey M, Cumulative radiation exposure from diagnostic imaging in intensive care unit patients. World J Radiol. 2016;8(4):419-427. doi:10.4329/wjr.v8.i4.419 27158429 PMC4840200

[45] Radiological Society of North America Radiation dose in x-ray and CT exams. 2017 https://www.radiologyinfo.org/en/info.cfm?pg=safety-xray. Accessed December 13, 2017.

[46] Wunsch H, Kramer A, Gershengorn HB Validation of intensive care and mechanical ventilation codes in Medicare data. Crit Care Med. 2017;45(7):e711-e714. doi:10.1097/CCM.0000000000002316 28403118 PMC6557134

