# Supplementary material for: Trends in Use of Daily Chest Radiographs Among US Adults Receiving Mechanical Ventilation
Source: JAMA Netw Open. 2018 Aug 10;1(4):e181119. doi: 10.1001/jamanetworkopen.2018.1119 (PMC6324260; doi:10.1001/jamanetworkopen.2018.1119)
Supplement: Supplement. — eTable 1. List of Charge Codes Used to Identify Chest Radiographs and Chest Tubes eTable 2. Factors Independently Associated With Receiving a Chest Radiograph Every Day (up to 7 days) Following Mechanical Ventilation Initiation eTable 3. Factors Associated With Being Admitted to Hospitals with the Highest vs Lowest Quartile of Use of Chest Radiographs Every Day (up to 7 days) Following Mechanical Ventilation Initiation eFigure 1. Pictogram of Cohort Creation and Outcome Assessment eFigure 2. Adjusted Percentage of Patients Receiving a Chest Radiograph Every Day (up to 7 days) Following Mechanical Ventilation Initiation by Discharge Quarter Stratified by Hospital Size and Geographic Region eFigure 3. Crude and Piecewise Regression-based Predicted Percentage of Patients Receiving a Chest Radiograph Every Day (up to 7 days) Following Mechanical Ventilation Initiation by Discharge Quarter eMethods. Methods for Estimating Hospital Cost Savings Associated with Reduced Daily CXR Use Across the US eReference [file jamanetwopen-1-e181119-s001.pdf]

Supplementary Online Content

Gershengorn HB, Wunsch H, Scales DC, Rubenfeld GD. Trends in use of daily chest radiographs among US adults receiving mechanical ventilation. *JAMA Netw Open*. 2018;1(4):e181119. doi:10.1001/jamanetworkopen.2018.1119

eTable 1. List of Charge Codes Used to Identify Chest Radiographs and Chest Tubes.

eTable 2. Factors Independently Associated With Receiving a Chest Radiograph Every Day (up to 7 days) Following Mechanical Ventilation Initiation.

eTable 3. Factors Associated With Being Admitted to Hospitals with the Highest vs Lowest Quartile of Use of Chest Radiographs Every Day (up to 7 days) Following Mechanical Ventilation Initiation.

eFigure 1. Pictogram of Cohort Creation and Outcome Assessment.

eFigure 2. Adjusted Percentage of Patients Receiving a Chest Radiograph Every Day (up to 7 days) Following Mechanical Ventilation Initiation by Discharge Quarter Stratified by Hospital Size and Geographic Region.

eFigure 3. Crude and Piecewise Regression-based Predicted Percentage of Patients Receiving a Chest Radiograph Every Day (up to 7 days) Following Mechanical Ventilation Initiation by Discharge Quarter.

eMethods. Methods for Estimating Hospital Cost Savings Associated with Reduced Daily CXR Use Across the US.

eReference

This supplementary material has been provided by the authors to give readers additional information about their work.

© 2018 Gershengorn HB. *JAMA Network Open*.

eTable 1. List of Charge Codes Used to Identify Chest Radiographs and Chest Tubes

Chest Radiographs	Chest Tubes
XR CHEST 1 VIEW	RN CHEST TUBE INSERTION
XR CHEST 1 VIEW PA	RN CHEST TUBE REMOVE
XR CHEST 1 VIEW PORTABLE	BAG CHEST DRAINAGE
XR CHEST DECUB 1 VIEW	BOTTLE CHEST DRAIN DOUBLE
XR CHEST 1 VIEW AP	BOTTLE CHEST DRAIN UNDERWATER
XR CHEST LORDOTIC 1 VIEW	CART CHEST TUBE
XR CHEST STEREO	CLAMP CHEST TUBE
XR CHEST 2 VIEWS	DRAIN CHEST DOUBLE
XR CHEST DECUB 2 VIEWS	DRAIN CHEST SETUP
XR CHEST OBLIQUE 2 VIEWS	DRAIN CHEST SINGLE
XR CHEST FLAT & UPRIGHT	DRAIN CHEST SINGLE INFANT
XR CHEST 2 VIEWS PORTABLE	DRAIN CHEST THORAKLEX
XR CHEST 2 VIEWS W/APICAL LORDOTIC	DRAIN HEIMLICH VALVE CHEST
XR CHEST W/OBLIQUES	KIT DRAIN CHEST UNDERWATER DRAIN BOTTLE
XR CHEST 2 VIEWS W/FLUORO	TRAY CHEST TUBE INSERTION
XR CHEST COMPLETE 4+ VIEWS	TROCAR CHEST TUBE
XR CHEST COMPLETE 4+ VIEWS W/FLUORO	DRAIN CHEST ATRIUM
XR CHEST SPECIAL VIEWS	THORACENTESIS W/INSERTION CHEST TUBE
	FIBRINOLYSIS VIA CHEST TUBE/CATHETER 1ST DAY
	FIBRINOLYSIS VIA CHEST TUBE/CATHETER EA SUB DAY
	CLOSURE CHEST POST DRAINAGE
	ER THORACENTESIS W/INSERTION CHEST TUBE
	ER FIBRINOLYSIS VIA CHEST TUBE/CATHETER 1ST DAY
	ER FIBRINOLYSIS VIA CHEST TUBE/CATHETER EA SUB DAY
	THORACENTESIS W/INSERTION CHEST TUBE OP
	PF THORACENTESIS W/INSERTION CHEST TUBE

© 2018 Gershengorn HB. *JAMA Network Open*.

	PF FIBRINOLYSIS VIA CHEST TUBE/CATHETER 1ST DAY
	PF FIBRINOLYSIS VIA CHEST TUBE/CATHETER EA SUB DAY

© 2018 Gershengorn HB. *JAMA Network Open*.

eTable 2. Factors Independently Associated With Receiving a Chest Radiograph Every Day (up to 7 days) Following Mechanical Ventilation Initiation

Covariate	Odds-Ratio (95% CI)	p-value[a]
Age (years)		
<50	1	
50-64	0.99 (0.97,1.01)	0.22
65-84	1.05 (1.03,1.08)	<0.001
85+	1.08 (1.05,1.11)	<0.001
Female	0.93 (0.92,0.94)	<0.001
Race		
White	1	
Black	0.84 (0.82,0.85)	<0.001
Other	0.92 (0.90,0.94)	<0.001
Insurance		
Private	1	
Medicare	0.86 (0.85,0.88)	<0.001
Medicaid	0.85 (0.83,0.86)	<0.001
Other/unknown	0.99 (0.97,1.02)	0.70
# of Elixhauser comorbidities	1.00 (0.99,1.00)	0.050
Major surgery	1.28 (1.26,1.31)	<0.001
Major Diagnostic Category		
can't be assigned	1	
nervous system	0.92 (0.90,0.95)	<0.001
eye	0.81 (0.50,1.29)	0.37
ear, nose, and throat	1.00 (0.90,1.11)	1.00
respiratory	1.44 (1.41,1.47)	<0.001
cardiovascular	1.89 (1.84,1.94)	<0.001
gastrointestinal (non-liver)	1.06 (1.02,1.09)	0.001
liver	1.23 (1.17,1.29)	<0.001
musculoskeletal	1.47 (1.40,1.55)	<0.001
dermatologic	0.77 (0.69,0.86)	<0.001
endocrine	1.24 (1.15,1.32)	<0.001
renal	0.92 (0.87,0.97)	0.001
genitourinary -male	0.94 (0.72,1.24)	0.66
genitourinary -female	1.21 (1.01,1.44)	0.037
obstetric	2.17 (1.89,2.49)	<0.001
neonatal		
hematologic	0.71 (0.64,0.79)	<0.001
myeloproliferative	1.47 (1.30,1.66)	<0.001
infectious	1.27 (1.24,1.30)	<0.001
psychiatric	1.24 (0.89,1.73)	0.21
ethanol/drug use	1.22 (1.12,1.34)	<0.001

© 2018 Gershengorn HB. *JAMA Network Open*.

injuries/poisonings	1.73 (1.67,1.80)	<0.001
burns	1.99 (1.57,2.53)	<0.001
health status influences	0.73 (0.60,0.90)	0.002
multiple trauma	2.10 (1.98,2.24)	<0.001
HIV	1.59 (1.46,1.74)	<0.001
Discharge quarter[b]	0.98 (0.98,0.98)	<0.001
Hospital bed #		
500+	1	
400-499	1.04 (0.74,1.44)	0.84
300-399	1.18 (0.87,1.60)	0.29
200-299	1.10 (0.82,1.48)	0.53
100-199	1.02 (0.75,1.40)	0.89
<100	1.46 (0.79,2.70)	0.23
Teaching hospital	1.01 (0.81,1.26)	0.90
Urban hospital	0.82 (0.63,1.07)	0.14
Geographic region		
Midwest	1	
Northeast	0.95 (0.70,1.28)	0.72
South	1.12 (0.88,1.42)	0.37
West	1.01 (0.74,1.36)	0.97
Median Odds-Ratio	2.43 (2.29,2.59)	

CI: confidence interval; HIV: human immunodeficiency virus

a A likelihood ratio test was conducted for each categorical independent variable (comparing the base model presented above to a model without that independent variable) to assess for the statistical significance of adjusting for the covariate of interest in its entirety; the results are—age: p<0.001; race: p<0.001; insurance: p<0.001; major diagnostic category: p<0.001; hospital bed #: p=0.75; geographic region: p=0.60

b discharge quarter is entered into model as a simple linear predictor here as the multilevel model evaluating the piecewise association of discharge quarter and daily CXR use did not converge; this odds-ratio reflects the average quarterly odds of use over time and does not take into account the more complex relationship of discharge date and daily CXR use due to the introduction of new guidelines in December, 2011 (see eFigure 3)

© 2018 Gershengorn HB. *JAMA Network Open*.

eTable 3. Factors Associated With Being Admitted to Hospitals With the Highest vs Lowest Quartile of Use of Chest Radiographs Every Day (up to 7 days) Following Mechanical Ventilation Initiation

	Univariate Comparisons[a]		Multivariate Comparisons[b]	
	Lowest Quartile Hospitals, (%)	Highest Quartile Hospitals, (%)	OR (95% CI)—Highest vs Lowest Use	p-value
General Cohort Information				
% of patients with CXR every day, median (full range)	36.8 (11.9,49.6)	83.4 (76.9,96.8)		
# of hospitals, N (%)	104 (50.0)	104 (50.0)		
# of patients, N (%)	115,793 (49.7)	117,047 (50.3)		
Patient Characteristics				
Age				
<50	18.2	19.0	1	
50-64	31.4	31.4	0.92 (0.86,0.97)	0.005
65-84	41.9	42.0	0.88 (0.79,1.00)	0.042
85+	8.6	7.5	0.83 (0.65,1.06)	0.13
Female[c]	46.7	46.0	0.99 (0.96,1.02)	0.45
Race				
White	61.9	71.1	1	
Black	21.5	14.1	0.59 (0.41,0.83)	0.003
Other	16.6	14.9	0.79 (0.50,1.23)	0.29
Insurance				
Private	17.6	18.6	1	
Medicare	59.1	59.1	0.97 (0.85,1.10)	0.60
Medicaid	14.0	12.5	0.86 (0.72,1.04)	0.13
Other/unknown	9.4	9.7	0.94 (0.72,1.22)	0.64
# of Elixhauser comorbidities, median(IQR)	5 (3,6)	5 (3,6)	1.02 (0.97,1.06)	0.48
Major Surgery	29.1	29.7	1.03 (0.95,1.13)	0.47
Major Diagnostic Category				
can't be assigned	15.2	14.5	1	
nervous system	8.7	8.2	1.03 (0.87,1.21)	0.76
eye	0.0	0.0	1.26 (0.67,2.39)	0.47
ear, nose, and throat	0.3	0.3	1.04 (0.82,1.33)	0.74
respiratory	24.5	26.1	1.03 (0.89,1.20)	0.69
cardiovascular	11.9	12.4	1.05 (0.89,1.25)	0.57

© 2018 Gershengorn HB. *JAMA Network Open*.

gastrointestinal (non-liver)	4.5	4.9	1.08 (0.93,1.26)	0.30
liver	1.8	1.8	1.01 (0.86,1.18)	0.93
musculoskeletal	1.7	1.7	1.02 (0.88,1.19)	0.78
dermatologic	0.3	0.3	0.87 (0.70,1.07)	0.20
endocrine	0.9	0.9	1.20 (1.01,1.43)	0.04
renal	1.5	1.4	0.93 (0.78,1.11)	0.43
genitourinary -male	0.0	0.0	1.01 (0.67,1.54)	0.95
genitourinary -female	0.1	0.1	0.86 (0.60,1.22)	0.39
obstetric	0.2	0.2	1.15 (0.84,1.58)	0.39
neonatal	0.3	0.3	1.04 (0.82,1.32)	0.74
hematologic	0.3	0.2	0.91 (0.69,1.21)	0.54
myeloproliferative	22.1	20.5	0.95 (0.80,1.11)	0.50
infectious	0.0	0.0	1.37 (0.73,2.55)	0.33
psychiatric	0.4	0.6	1.36 (1.02,1.82)	0.04
ethanol/drug use	3.2	3.8	1.17 (0.97,1.40)	0.09
injuries/poisonings	0.0	0.1	2.21 (0.30,16.39)	0.44
burns	0.1	0.1	0.84 (0.58,1.22)	0.35
health status influences	1.3	1.3	1.01 (0.68,1.50)	0.97
multiple trauma	0.6	0.3	0.83 (0.64,1.08)	0.17
Discharge year[d]			0.99 (0.97,1.00)	0.12
2008	5.3	6.2		
2009	13.6	14.9		
2010	14.1	15.6		
2011	16.2	16.0		
2012	16.5	16.3		
2013	17.9	17.0		
2014	16.6	14.1		
Hospital Characteristics				
Hospital bed #				
500+	35.7	28.1	1	
400-499	15.8	11.6	0.79 (0.24,2.56)	0.69
300-399	19.2	27.9	1.47 (0.47,4.62)	0.51
200-299	18.8	25.1	1.24 (0.44,3.52)	0.68
100-199	10.3	6.1	0.44 (0.12,1.54)	0.20
<100	0.3	1.2	2.94 (0.27,32.10)	0.38
Teaching hospital	53.4	39.6	0.73 (0.31,1.68)	0.46
Urban hospital	93.8	86.2	0.45 (0.18,1.11)	0.08
Geographic region				
Midwest	14.1	21.0	1	
Northeast	21.4	7.6	0.27 (0.07,0.96)	0.04

© 2018 Gershengorn HB. *JAMA Network Open*.

South	49.6	53.6	0.69 (0.25,1.95)	0.49
West	14.8	17.8	0.67 (0.20,2.22)	0.51
Outcomes				
MV duration, median(IQR)	5 (3,9)	5 (3,9)		
Hospital length of stay (days), median(IQR)	12 (7,20)	12 (7,19)		
Hospital mortality	27.7	25.6		

CI: confidence interval; CXR: chest radiograph; IQR: interquartile range; HIV: human immunodeficiency virus; MV: mechanical ventilation

[a]All comparisons by "everyday CXR" status, p≤0.005 (Chi Square testing for categorical; Kruskal-Wallis testing for continuous variables)
[b]Multilevel model did not converge; instead, used single-level multivariable logistic regression model with clustering of standard errors by individual hospital
[c]Missing from 47 (0.009%) of cohort
[d]For multivariable model, included discharge quarter as a continuous variable.

© 2018 Gershengorn HB. *JAMA Network Open*.

eFigure 1. Pictogram of Cohort Creation and Outcome Assessment

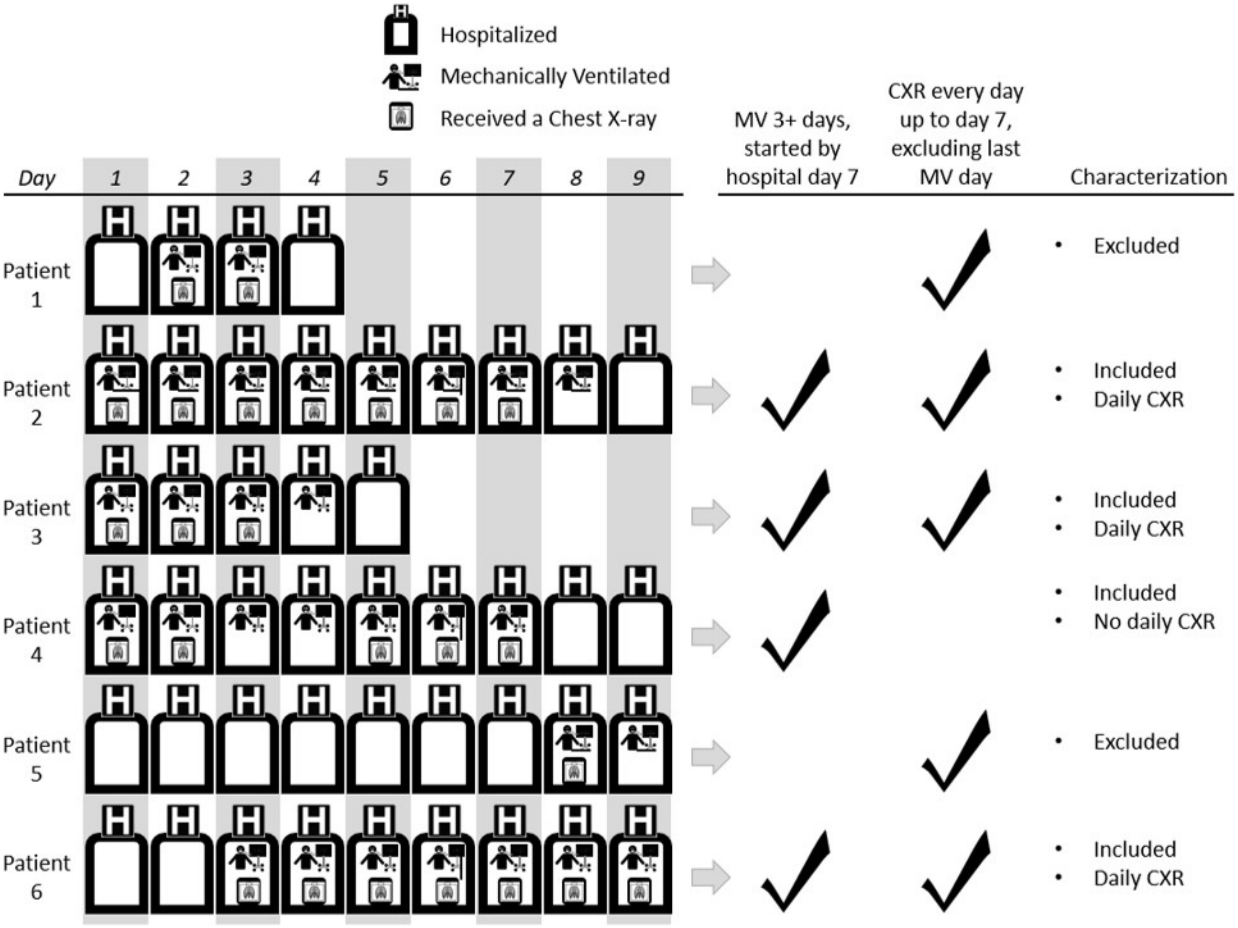

CXR: chest radiograph; MV: mechanical ventilation. Patient 1—excluded because not MV for 3+ days; patient 2—included as MV for 8 days and receiving a daily CXR as had one for 7 of the first 7 days of MV; patient 3— included as MV for 4 days and receiving a daily CXR as had one for the first 3 of the 4 days of MV (last day is excluded from "daily" calculation); patient 4— included as MV for 7 days and not receiving a daily CXR as did not receive one on days 3 and 4 of MV; patient 5— excluded because MV started after hospital day 7 (and not for 3+ days); patient 6— included as MV for 7 days and receiving a daily CXR as had one for 7 of the first 7 days of MV

© 2018 Gershengorn HB. *JAMA Network Open*.

eFigure 2. Adjusted Percentage of Patients Receiving a Chest Radiograph Every Day (up to 7 days) Following Mechanical Ventilation Initiation by Discharge Quarter Stratified by Hospital Size and Geographic Region

A. Hospital Bed Number

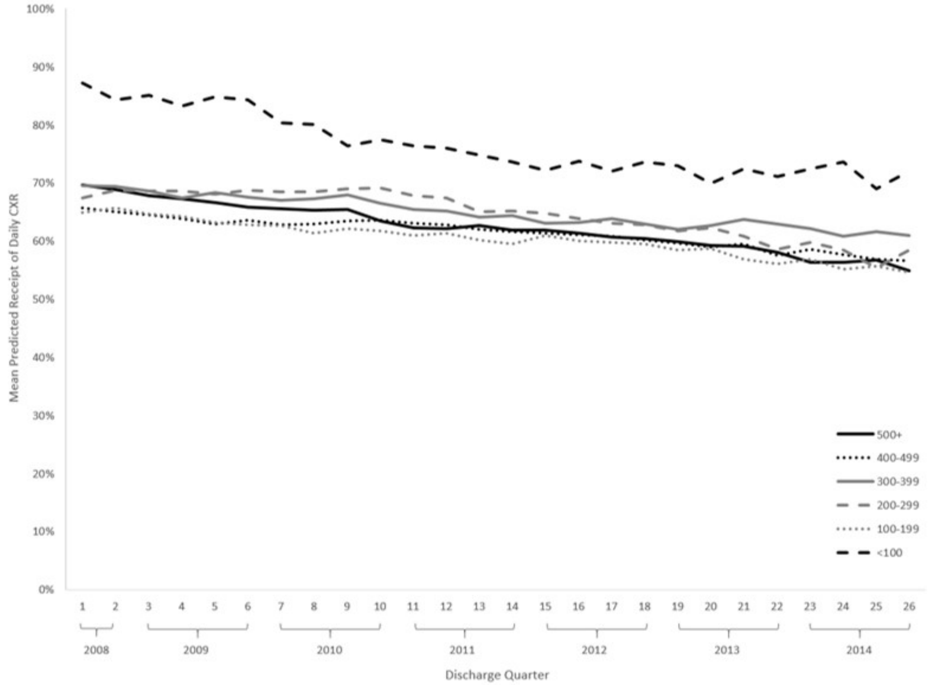

B. Geographic Region

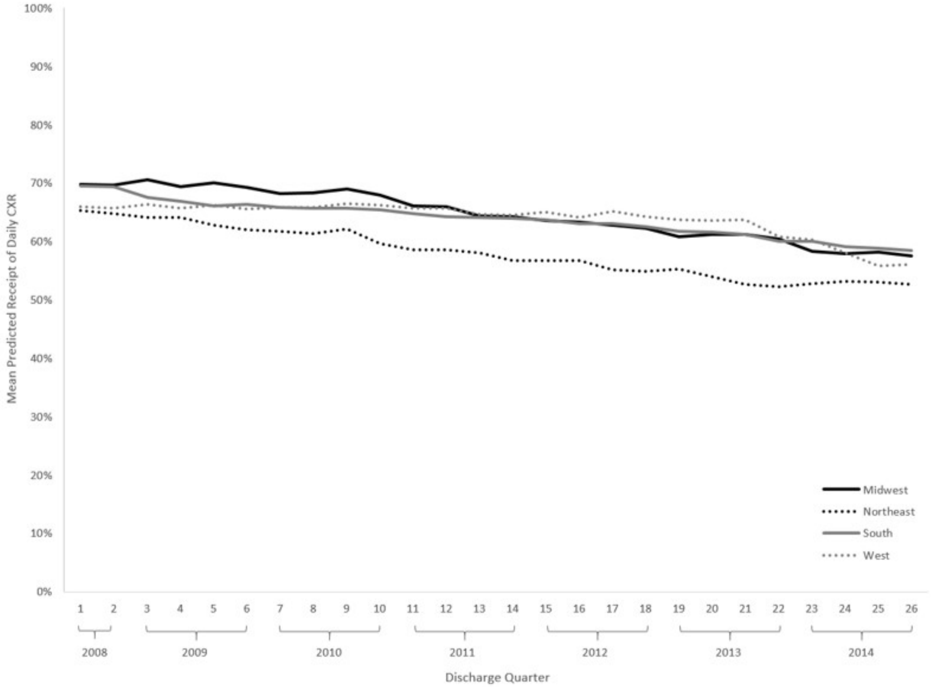

CXR: chest radiograph; MV: mechanical ventilation. Results from the primary multilevel multivariable model (without interaction terms) for hospital-level factors found to have an interaction with discharge quarter—bed number, p<0.05 for all groups; geographical

© 2018 Gershengorn HB. *JAMA Network Open*.

region, p<0.001 for all groups—are presented. Teaching status (p=0.17) and urban community (p=0.35) did not demonstrate a significant interaction with discharge quarter and are not shown here; the model with an interaction term including urban location was single level (with clustering of standard errors by individual hospital as the multilevel model did not converge). The "mean predicted receipt of a daily CXR" was calculated as the average of the predicted probabilities of obtaining a daily CXR for all patients within each discharge quarter and each hospital-level factor strata (e.g., hospital bed number, geographic region).

© 2018 Gershengorn HB. *JAMA Network Open*.

eFigure 3. Crude and Piecewise Regression-based Predicted Percentage of Patients Receiving a Chest Radiograph Every Day (up to 7 days) Following Mechanical Ventilation Initiation by Discharge Quarter

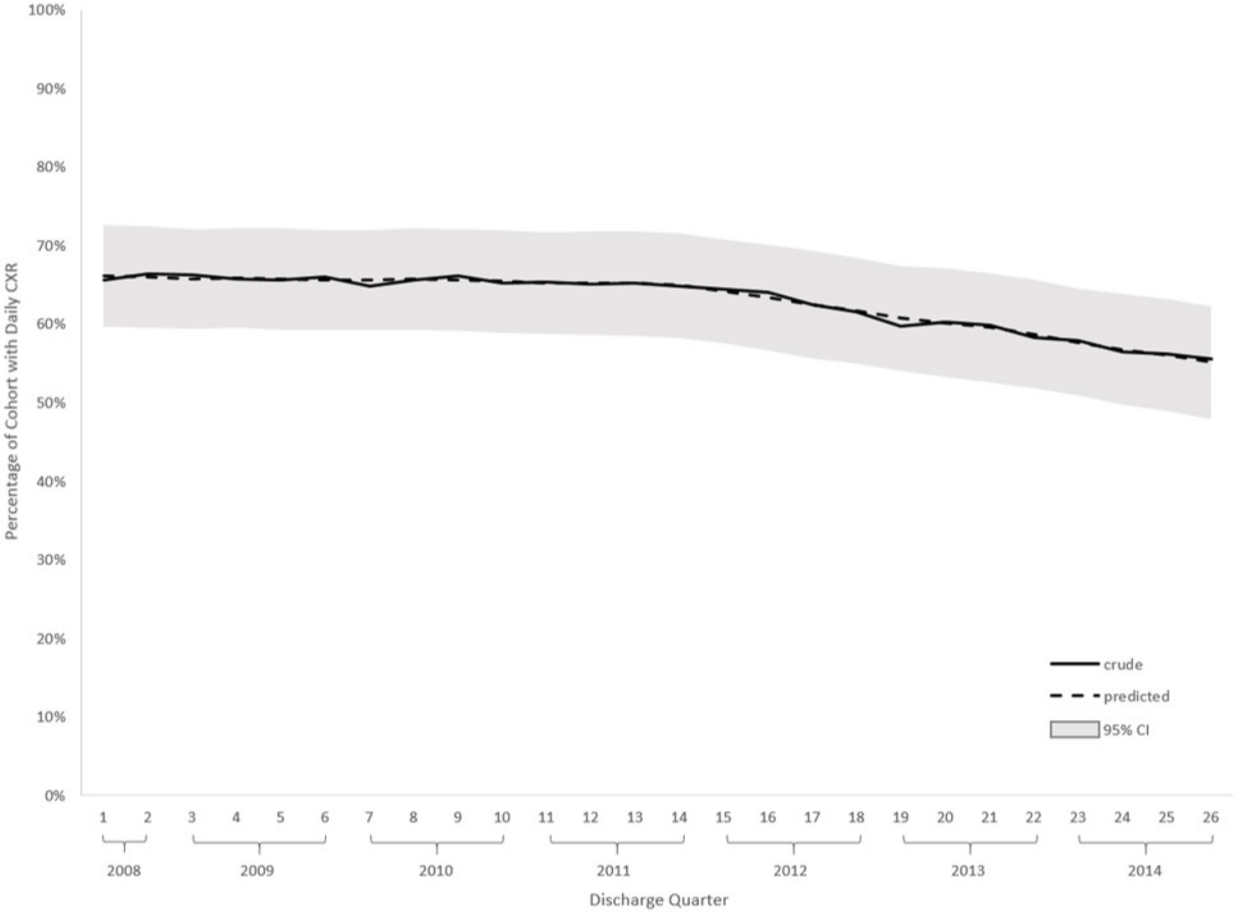

CI: confidence interval; CXR: chest radiograph; MV: mechanical ventilation. The predicted percentage of patients receiving a daily CXR was calculated as the average of the predicted probabilities of obtaining a daily CXR for all patients within each discharge quarter using the piecewise regression-based model.

© 2018 Gershengorn HB. *JAMA Network Open*.

eMethods. Methods for Estimating Hospital Cost Savings Associated with Reduced Daily CXR Use Across the US

Assumptions:

1. all hospitals in the highest three quartiles of daily CXR use (n=312) instead performed daily CXRs only as often as the hospital with the highest rate of daily CXR use in the lowest quartile of everyday use; and,
2. these high use hospitals instead performed CXRs on 50% of MV days (rather than 100%) for the patients who received CXRs every day at their hospital.

Calculation steps:

1. Identify the % of patients in each hospital (*h*) in the highest 3 quartiles of everyday CXR use that would no longer get a daily CXR ($\Delta P_{CXR\_use,h}$) if their hospital performed daily CXRs at the rate of the hospital with the highest rate of daily CXR use in the lowest quartile of use ($P_{benchmark\_use}$= 49.6%, rounded to 50%):

$$\Delta P_{CXR\_use,h} = P_{current\_use,h} - P_{benchmark\_use} = P_{current\_use,h} - 50\%$$

$P_{current\_use,h}$ = current rate of daily CXRs at hospital *h*

2. Determine the number of patients this would impact in each hospital ($N_{impacted,h}$):

$$N_{impacted,h} = N_h * \Delta P_{CXR\_use,h}$$

N_h = # of MV patients in hospital *h*

3. Determine the number of CXRs "saved" ($CXR_{saved,h}$) assuming daily CXRs cut to ½ of all days of MV (up to day 7; $D_{everyday\_pts,h}$) for these impacted patients:

$$CXR_{saved,h} = \sum_{h=1}^{n} (N_{impacted,h} * D_{everyday\_pts,h} * ½)$$

4. Determine the estimated cost savings across cohort hospitals (CS_{cohort}) using data from the 2014 charge master (median hospital costs: $60 for technical; $0.75 for professional CXR services):

$$CS_{cohort} = CS_{tech} + CS_{prof} = \$60 * CXR_{saved,h} + \$0.75 * CXR_{saved,h}$$

CS_{tech} = cost savings from technical services in cohort hospitals
CS_{prof} = cost savings from professional services in cohort hospitals

5. Extrapolate cost savings from included cohort hospitals (n=416) to total cost savings (CS_{total}) for all acute care hospitals in 2014 in the US which were likely to care for MV patients (H_{US})[1]:

© 2018 Gershengorn HB. *JAMA Network Open*.

H_{US} = American Hospital Association registered hospitals (total n=5,723) which are not primarily psychiatric (n=413), long term care (n=89), or institution-based (n=11) = 5,210

$$CS_{total} = \frac{5,210}{416} * CS_{cohort} \qquad CS_{total,tech} = \frac{5,210}{416} * CS_{tech} \qquad CS_{total.prof} = \frac{5,210}{416} * CS_{prof}$$

$CS_{total,tech}$ = cost savings from technical services in US hospitals
$CS_{total,prof}$ = cost savings from professional services in US hospitals

© 2018 Gershengorn HB. *JAMA Network Open*.

eReference

1. American Hospital Association. Fast Facts on US Hospitals. 2014; http://www.aha.org/research/rc/stat-studies/fast-facts2014.shtml. Accessed January 2, 2014, 2017.

© 2018 Gershengorn HB. *JAMA Network Open*.
